# Adenoma location, size, and morphology are risk factors for FOBT false-negative results in inpatients with advanced colorectal adenoma

**DOI:** 10.1038/s41598-024-51377-0

**Published:** 2024-01-08

**Authors:** Xu Cao, Ping Meng, Yong Liu, Xiaofang Li, Xiaoyang Shi, Xiaoxing Sun, Tianpeng Zhang, Jinfeng Wang, Hao Jiao, Huijie Wang, Huanwei Zheng

**Affiliations:** 1 Department of Endoscopy, Shijiazhuang Traditional Chinese Medicine Hospital, Shijiazhuang, 050000 China; 2 Department of Gastroenterology, Shijiazhuang Traditional Chinese Medicine Hospital, Shijiazhuang, 050000 China; 3 Department of Anorectum, Shijiazhuang Traditional Chinese Medicine Hospital, Shijiazhuang, 050000 China; 4 Department of Surgery, Shijiazhuang Traditional Chinese Medicine Hospital, Shijiazhuang, 050000 China

**Keywords:** Intestinal diseases, Gastrointestinal cancer

## Abstract

Recently, advanced adenoma (AA) has been recognized as a target for colorectal cancer (CRC) screening. However, the fecal occult blood test (FOBT), the primary non-invasive screening method, shows limited sensitivity in detecting AA. This study investigates the relationship between adenoma characteristics and FOBT false-negative results. In a retrospective cohort study conducted from 2015 to 2022, we examined 342 inpatients with AA who underwent colonoscopy and received qualitative FOBT. FOBT sensitivity was analyzed about various adenoma characteristics, and logistic regression models were employed to investigate the relationship between adenoma features and FOBT false-negative outcomes. FOBT sensitivity in AA inpatients was 52.63%. Significant differences in sensitivity were observed based on adenoma location (left vs. right), morphology (with or without pedunculation), and size (≤ 10 mm vs. > 10 mm). After adjusting for several potential confounders, FOBT showed a reduced false-negative rate in AA with large-sized (OR, 0.49; 95% CI 0.31–0.77), left-sided location (OR, 0.53; 95% CI 0.31–0.89), and pedunculated morphology (OR, 0.73; 95% CI 0.43–1.24). AA with large size, left-sided location, and pedunculated morphology independently contribute to a decreased rate of FOBT false-negative results. However, these adenoma characteristics are not actively modifiable. Therefore, novel non-invasive methods are needed to improve AA detection accuracy.

## Introduction

In 2020, colorectal cancer (CRC) ranked as the third most prevalent malignancy worldwide and the second leading cause of cancer-related mortality, posing a significant burden on healthcare systems^1,2^. Given the challenges associated with implementing significant lifestyle changes or comprehensive primary prevention strategies to reduce CRC risk, screening has emerged as the most effective public health tool for reducing mortality rates^3^. Recently, the World Endoscopy Organization issued an update evaluating validated strategies for novel non-invasive screening tests for CRC, recognizing precursor lesions for CRC as legitimate screening targets^4^. In the adenoma-cancer sequence, advanced adenoma (AA) represents the highest risk stage of precancerous lesions^5^. AA is defined as an adenoma with at least one of the following characteristics: size ≥ 1 cm, tubulovillous or villous components, or high-grade dysplasia.

In China, several prominent CRC screening programs and the majority of hospitals incorporate qualitative fecal occult blood test (FOBT) into their screening protocols^6–8^. FOBT is widely accepted as a non-invasive CRC screening method^9^ owing to its ease of administration and its proven efficacy in reducing CRC-related mortality^10^. However, the effectiveness of FOBT is inherently linked to achieving high sensitivity and compliance rates^11^. Unfortunately, FOBT displays suboptimal sensitivity when applied to AA^12^.

Regarding the phenomenon of FOBT false-negative outcomes, a comprehensive meta-analysis has revealed a spectrum of individual factors, including male sex, hyperglycemia, hypertension, obesity, and smoking, that increase the risk of obtaining false-negative results among individuals afflicted by advanced colorectal neoplasms, encompassing both AA and CRC^13^. Moreover, the accuracy of FOBT depends on various determinants, such as tumor location, size, morphology, and other lesion-specific attributes^11,14,15^. While a limited number of studies have explored the connection between these characteristics and the occurrence of FOBT false-negative outcomes, the findings are inconsistent, and the identification of independent risk factors remains elusive^11,16^, particularly in the realm of histological classifications. This understanding is of paramount importance for screening initiatives that aim to preemptively identify high-risk adenomas and individualize strategies for colonoscopy interventions and treatment^17^. The overarching objective of abating CRC-associated mortality and morbidity can be more efficaciously pursued through the implementation of a robust AA screening paradigm.

Given the scarcity of investigations into the interplay between AA features and FOBT false-negative outcomes, we embarked on this retrospective inquiry with the principal aim of appraising the qualitative FOBT sensitivity across diverse adenoma attributes in hospitalized individuals diagnosed with AA. Simultaneously, we endeavored to pinpoint adenomatous traits that independently contribute to the occurrence of FOBT false-negative results.

## Results

### Participants

A total of 342 patients with AA were included in this study. Participant characteristics were stratified according to FOBT false-negative results, as summarized in Table 1. Three key indicators, including the location, size, and pedunculated type of AA, exhibited significant differences between the two groups and were further analyzed using multivariable logistic regression. We also analyzed adenoma characteristics and demographic variables in those with and without FOBT data, which showed no significant differences except for the drinking status (Table S1).

**Table 1:** Participants characteristics.

Variables	Total n = 342	False-negative n = 162	True-positive n = 180	*P*-values
Age (year)	61.9 ± 9.7	62.5 ± 8.8	61.5 ± 10.5	0.351
Sex, male, n (%)	221 (64.6)	107 (66)	114 (63.3)	0.600
Weight (kg)	70.0 ± 11.7	69.6 ± 11.4	70.4 ± 12.0	0.540
Marital status, n (%)				0.723
Single/divorced	12 (3.5)	5 (3.1)	7 (3.9)	
Married	312 (91.2)	147 (90.7)	165 (91.7)	
Others	18 (5.3)	10 (6.2)	8 (4.4)	
Smoking status, n (%)				0.922
Non-smoker	207 (60.5)	108 (60)	99 (61.1)	
Current-smoker	34 (9.9)	19 (10.6)	15 (9.3)	
NA	101 (30.1)	53 (29.9)	48 (30.2)	
Drinking status, n (%)				0.979
Non-drinker	203 (59.4)	106 (58.9)	97 (59.9)	
Current-drinker	39 (11.4)	21 (11.7)	18 (11.1)	
NA	100 (29.2)	53 (29.4)	47 (29)	
Family history of CRC, n (%)	4 (1.2)	1 (0.6)	3 (1.7)	0.625
Antiplatelet anticoagulant use, n (%)	40 (11.7)	18 (11.1)	22 (12.2)	0.750
Laboratory indicators				
PLT, × 10^9^/L	219.3 ± 62.6	216.2 ± 52.9	222.1 ± 70.2	0.771
HGB (g/L)	138.9 ± 19.0	140.2 ± 16.8	137.8 ± 20.7	0.255
TG (mmol/L)	1.5 (1.1, 2.0)	1.4 (1.0, 1.8)	1.5 (1.1, 2.2)	0.179
TC (mmol/L)	4.8 ± 1.1	4.7 ± 1.0	4.8 ± 1.2	0.449
HDL (mmol/L)	1.3 ± 0.3	1.3 ± 0.3	1.3 ± 0.3	0.980
LDL (mmol/L)	2.8 ± 0.7	2.8 ± 0.6	2.8 ± 0.7	0.309
Comorbidities, n (%)				
Hypertension	138 (40.4)	63 (38.9)	75 (41.7)	0.601
Ischemic cerebrovascular disease	53 (15.5)	28 (17.3)	25 (13.9)	0.386
Coronary heart disease	57 (16.7)	28 (17.3)	29 (16.1)	0.771
HLP	43 (12.6)	22 (13.6)	21 (11.7)	0.594
Liver disease	40 (11.7)	17 (10.5)	23 (12.8)	0.512
DM	55 (16.1)	26 (16)	26 (16)	0.988

Data are presented as the N (%), median (quartile 1–quartile 3), or mean ± SD.

*PLT* platelets, *HGB* hemoglobin, *TG* triglyceride, *TC* total cholesterol, *HDL* high-density lipoprotein, *LDL* low-density lipoprotein, *HLP* hyperlipidemia, *DM* diabetes mellitus, *NA* not recorded.

### Sensitivity of FOBT in different advanced adenoma characteristics

At the manufacturer's recommended FOBT threshold, the overall participants’ cohort exhibited a sensitivity of 52.63%. Importantly, FOBT sensitivity in patients with AA revealed noteworthy disparities by adenoma location (left and right), morphology (pedunculated or non-pedunculated), and size (≤ 10 mm and > 10 mm). Notably, there were notable decreases in FOBT sensitivity for AA located on the right side, those lacking a pedunculated configuration, and those measuring ≤ 10 mm in size. However, in contrast, statistical variances in FOBT sensitivity were not observed between the combined non-AA group and distinct histological type groups (with villous and high-grade components). For details see Table 2.

**Table 2:** Sensitivity of FOBT in different advanced adenoma characteristics.

Adenoma characteristics	Total, n	False-negative	True-positive	Sensitivity (%)	*P*-values
Total	342	162	180	52.6 (47.2–58.0)	
Size					0.005
≤ 10 mm	148	83	65	43.9 (35.8–52.3)	
> 10 mm	194	79	115	59.3 (52.0–66.3)	
Location					0.001
Right-sided	106	64	42	39.6 (30.3–49.6)	
Left-sided	236	98	138	58.5 (51.9–64.8)	
Pedunculated type					0.021
No	248	127	121	48.8 (42.4–55.2)	
Yes	94	35	59	62.8 (52.2–72.5)	
With non-advanced adenoma					0.738
No	36	18	18	50.00 (32.9–67.1)	
Yes	306	144	162	52.9 (47.2–58.6)	
With villous component					0.256
No	263	129	134	51.0 (44.7–57.1)	
Yes	79	33	46	58.2 (46.6–69.2)	
With high-grade dysplasia					0.080
No	299	147	152	50.8 (45.0–56.6)	
Yes	43	15	28	65.1 (49.1–79.0)	

Sensitivity = true-positive/(true-positive + false-negative).

### Relationship between the FOBT false-negative results with advanced colorectal adenoma

Our investigation into the relationship between FOBT false-negative results and AA involved an exhaustive analysis of adenoma location, size, and pedunculated type (Table 3), conducted through multivariable logistic regression analyses. Our findings underscored the robust nature of the connections between FOBT false-negative results and the location, size, and pedunculated type of AA.

**Table 3:** Multivariable logistic regression analyses between the size, location, and pedunculated type of advanced adenoma with the false negative results of the FOBT.

Variable	Event, n	Crude model^a^		Model 1^b^		Model 2^c^	
		OR 95% CI	*P*-value	OR 95% CI	*P*-value	OR 95% CI	*P*-value
Size, mm	162/342 (47.4)	0.43 (0.28–0.67)	< 0.001	0.43 (0.28–0.66)	< 0.001	0.49 (0.31–0.77)	0.002
Location							
Right-sided	64/106 (60.4)	1 (Reference)		1 (Reference)		1 (Reference)	
Left-sided	98/236 (41.5)	0.47 (0.29–0.74)	0.001	0.47 (0.29–0.75)	0.001	0.53 (0.31–0.89)	0.016
Pedunculated type							
No	127/248 (51.2)	1 (Reference)		1 (Reference)		1 (Reference)	
Yes	35/94 (37.2)	0.57 (0.35–0.92)	0.022	0.56 (0.34–0.92)	0.021	0.73 (0.43–1.24)	0.246

*OR* odds ratio, *CI* confidence interval, *PLT* platelets, *HGB* hemoglobin, *TG* triglyceride, *CRC* colorectal cancer, *HLP* hyperlipidemia, *DM* diabetes mellitus.

^a^Unadjusted; ^b^Adjusted for sex and age; ^c^Adjusted for sex, age, weight, smoking, drinking, antiplatelet anticoagulant use, size, location, pedunculated type, high-grade dysplasia, villous component, TG, PLT, HGB, family history of CRC, hypertension, HLP, and DM except for the variable itself.

In the Crude Model, the association between size and FOBT false-negative results exhibited a negative correlation (OR: 0.43, 95% CI 0.28–0.67). Notably, even after accounting for a broader spectrum of potential covariates in Models 1 and 2, this association remained consistent (Model 1: OR, 0.43; 95% CI 0.28–0.6; Model 2: OR, 0.49, 95% CI 0.31–0.77). Furthermore, in all multivariable logistic regression models, compared with AA located in the right-sided colorectal or without pedunculated type, respectively, the left-sided AA or pedunculated type have a decreased risk of false-negative FOBT results. For instance, when compared with right-sided AA, the odds ratios (ORs) in the Crude Model, Model 1, and Model 2 were 0.47 (95% CI 0.29–0.74), 0.47 (0.29–0.75), and 0.53 (0.31–0.89), respectively. Similarly, when compared with adenomas without a pedunculated type, the ORs in the Crude Model, Model 1, and Model 2 were 0.57 (95% CI 0.35–0.92), 0.56 (0.34–0.92), and 0.73 (0.43–1.24), respectively.

Stratified analyses were executed to assess the potential influence of the relationship between size, location, and pedunculated type (Fig. S1) on FOBT false-negative results. Following stratification by sex, age (< 65 and ≥ 65 years), ischemic cerebrovascular disease, hypertension, hyperlipidemia (HLP), and diabetes mellitus (DM), no substantial interactions were identified. Notably, in the analysis exploring the association between AA size and FOBT false-negative results within the hypertensive subgroup, although the interaction *P* = 0.037, the result did not achieve statistical significance after accounting for multiple testing.

## Discussion

This retrospective study aimed to assess the sensitivity of qualitative FOBT in patients with AA and identify adenoma characteristics independently associated with false-negative results. As anticipated, our results, consistent with other studies, demonstrate a lower sensitivity of FOBT in detecting AA compared to the over 70% sensitivity confirmed in previous research for CRC^18^. Nevertheless, notable distinctions were also evident concerning the adenoma's location, size, and type. Specifically, FOBT sensitivity exhibited a decrease in right-sided adenomas, adenomas without pedunculated type, and adenomas ≤ 10 mm in size. Multivariate logistic regression analysis demonstrated a negative correlation between FOBT false-negative results and adenoma size, left-sided location, and pedunculated type. Surprisingly, histological type did not show a significant association with false-negative results.

Previous studies have examined the relationship between individual factors and FOBT false-negative results in patients with advanced colorectal tumors, encompassing AA and CRC. Kim et al.^16^ found that gender and smoking status were not associated with false-negative FOBT results in patients with advanced colorectal tumors; and elevated fasting glucose (adjusted odds ratio [AOR], 0. 59; 95% CI 0.36‒0.97) was associated with a lower risk of false-negative FOBT results. Wong et al.^19^ found that patients aged 66‒70 years with advanced colorectal tumors (AOR, 0. 31; 95% CI 0. 13‒0. 74) had the lowest likelihood of a false-negative FOBT result. In addition, a meta-analysis found that men (RR 1.83, CI 1.53‒2.19) with a family history of colorectal cancer (RR 1.61, CI 1.19‒2.15), hypertension (RR 1.50, CI 1.14‒1.98), and (ex-)smokers (RR 1.93, CI 1.52‒2.45) had a higher risk of false negative results^13^. To mitigate potential confounding effects on our analysis, we integrated these factors as covariates in our multivariable logistic regression models. However, these studies have often overlooked the differentiation between AA and CRC. Given the growing recognition of AA as a legitimate screening target^4^, it becomes imperative to scrutinize the risk factors associated with FOBT false-negative results specifically in the context of AA.

In a study conducted by Chiu et al.^11^, the investigation into the association between distinct adenoma characteristics and FOBT false-negative results in AA patients employed the concept of 'per-adenoma' analysis. Their findings indicated a greater likelihood of yielding false-negative results in cases of small lesions (measuring less than 15 mm) and lesions lacking a pedunculated type. These were consistent with our results. Surprisingly, Chiu et al. did not identify significant differences in adenoma location, a contrast to our findings. In a study that established an association between colonic lesions identified through the Florence Screening Program and fecal hemoglobin content assessed by FOBT, heightened fecal hemoglobin content displayed significant correlations with lesion size (*P* = 0.0000), severe heterogeneous hyperplasia (*P* = 0.0001), villous component (*P* = 0.0002), and left-sided localization (*P* = 0.003), as determined by univariate analysis^20^. Contrarily, our study did not identify a significant correlation between high-grade dysplasia and villous components, which are characteristics of adenomas, and false-negative FOBT results. These disparities may stem from differing FOBT thresholds or unaccounted factors influencing FOBT false-negative results in AA patients. These variables warrant further exploration in future studies.

Multiple factors may underlie the increased likelihood of FOBT false-negative results in patients with smaller or right-sided AA. The size of the lesion, to a certain extent, determines the extent of blood loss^17^, with smaller lesions usually resulting in less bleeding^21^ that may not meet the fixed threshold for quantitative FOBT. In addition, hemoglobin produced from left-sided tumors is less prone to degradation compared to tumors on the right-sided^22^. The firmer consistency of stool on the left side is more likely to trigger bleeding from the tumor during passage^14^. Pedunculated type adenomas are more prone to rubbing against fecal due to their morphological features, which increases the likelihood of causing bleeding^23^.

In the context of analyzing FOBT sensitivity across different sites of AA, certain studies have incorporated all right-sided tumors (some combined left-sided tumors) when evaluating the sensitivity of colorectal right-sided tumors (and vice versa)^24^. This approach would increase the sensitivity of screening right-sided tumors using FOBT, as some FOBT-positive results may arise from left-sided tumors. Conversely, studies akin to ours^25,26^ have exclusively considered the sensitivity of subjects with either left- or right-sided tumors, offering a more precise assessment of the correlation between tumor location and FOBT results.

Enhancing the detection of AA can significantly contribute to preventing CRC and reducing cancer-related mortality. Achieving this goal, as demonstrated in our study, hinges on understanding the risk factors for test errors in patients with AA, enabling the refinement of screening programs. Regrettably, the ability to proactively alter the risk factors linked to FOBT false-negative outcomes is limited, encompassing the adenoma characteristics highlighted in our study and the individual factors previously identified. Consequently, the future calls for the development of novel non-invasive screening methods to enhance the accuracy of AA detection.

Screening programs and clinical practices typically use a single FOBT to assess patients^8^. Numerous studies have analyzed whether the number of specimens would impact the sensitivity of FOBT in AA patients. The US Multi-Society Task Force on Colorectal Cancer reviewed reports related to the characteristics of FOBT and colorectal tumor detection tests in 2017^18^. They mentioned a study based on the Korean population, which used AA as the screening target. No difference was observed in the receiver operator characteristic curve with more FOBT samples, indicating that multiple samples are equivalent to one sample in terms of detecting AA^27^. Similarly, studies from Hong Kong^24^, France^28^, and Spain^29^ also found that the second set of test kits had no advantage in detecting advanced tumors. However, Rozen et al. found that the identification of colorectal adenoma through quantitative immunochemical FOBT depends on the number of examinations conducted^17^. Although the more tests analyzed per patient, the higher the sensitivity, the specificity for AA decreases. While sensitivity and specificity are crucial for achieving the goal of minimizing the burden of CRC, blindly improving sensitivity and reducing specificity is not an ideal approach. Therefore, future research needs to further explore and optimize the testing norms of FOBT, to more effectively screen and diagnose advanced adenomas while maintaining high sensitivity and improving specificity.

It is important to acknowledge the limitations of our study. Firstly, although qualitative FOBT is a commonly employed colorectal screening method in Chinese hospitals, the correlation between adenoma characteristics and fecal hemoglobin concentration could not be explored^30^. Further investigations are warranted to delve into the relationship. Secondly, another important limitation is that a proportion of the entire population of patients with AA lacks FOBT results, possibly because of poor compliance with FOBT. Although there were no significant differences in adenoma characteristics and demographic variables between the populations with or without FOBT except for the drinking status, potential bias due to population selection may affect the accuracy of results. Lastly, the absence of multiple FOBT results is noteworthy, as multiple tests are known to enhance sensitivity^31^.

## Methods

### Study population

**Figure 1:**
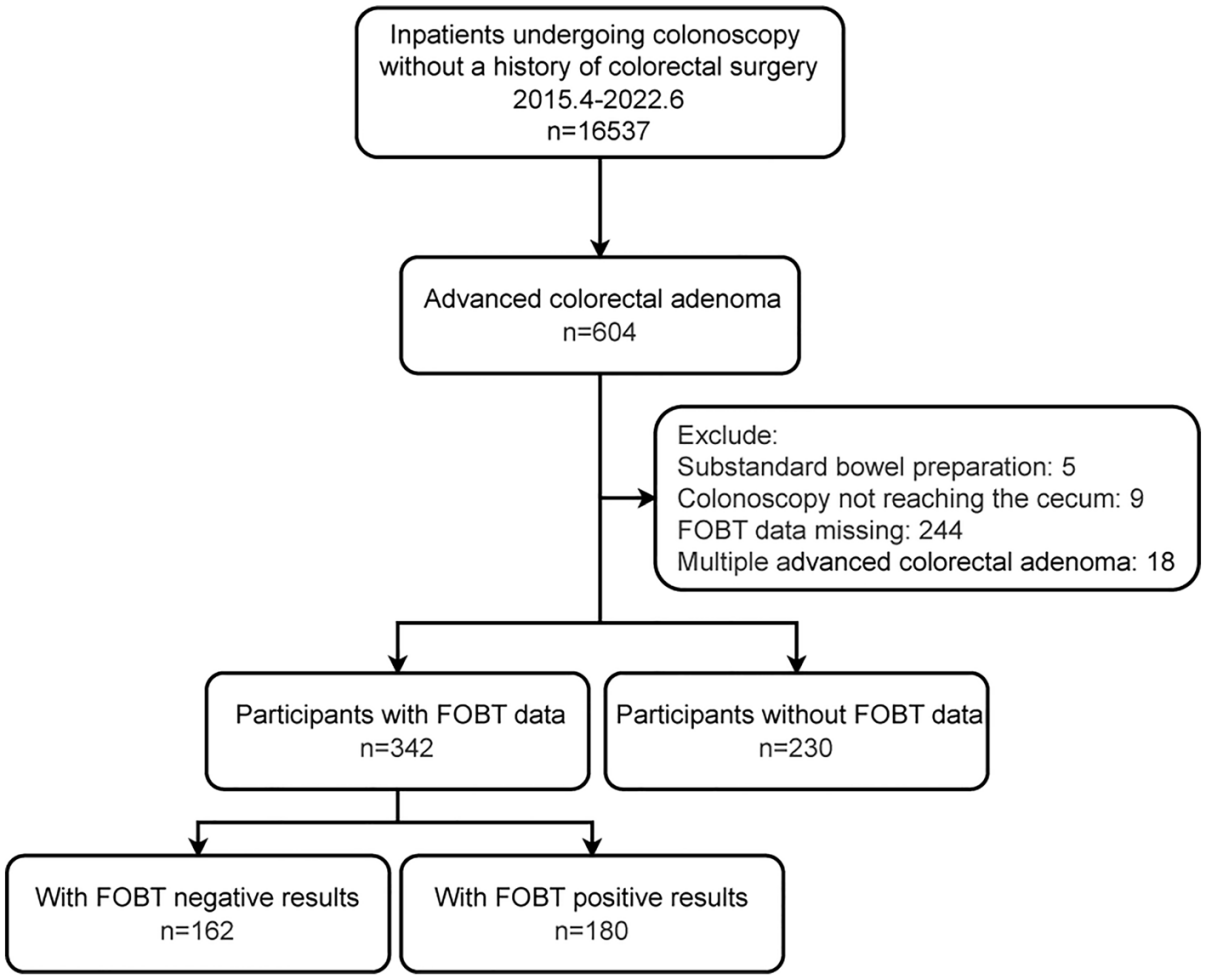
Flowchart of population.

In this retrospective study, we included consecutive inpatients who underwent colonoscopies at Shijiazhuang Traditional Chinese Medicine Hospital from April 2015 to June 2022. Each patient was enrolled only once, and the study cohort comprised individuals diagnosed with AA who had undergone FOBT. In the 604 AAs, we excluded the following: 5 cases with substandard bowel preparation, 9 cases where colonoscopy did not reach the cecum, 244 cases with missing FOBT data, and 18 cases with multiple AAs. Details are outlined in Fig. 1.

### Ethics approval

This retrospective study was approved by the Ethics Review Board of Shijiazhuang Traditional Chinese Medicine Hospital (No. 20220919029) and the requirement for informed consent was waived because of the retrospective nature of the study. All methods were performed in accordance with the relevant guidelines and regulations.

### Definition and covariates

AA is diagnosed through a combination of endoscopy and histopathology. A true-positive case was defined as a patient diagnosed with AA with a positive FOBT result; a false-negative case was defined as an individual diagnosed with AA but with a negative FOBT result.

The classification of colorectal tumor location was determined based on anatomical distribution, with the inclusion of the colorectum below the splenic flexure, including the splenic flexure itself, being designated as left-sided^32^. The FOBT kit, employed in this study, is manufactured by Sichuan Orienter Biotechnology Co., Ltd. It utilizes the colloidal gold immunochromatographic technique with a double antibody sandwich configuration. For sample collection, fresh fecal matter is required. The clean sample collection tube is unsealed, and the sampling spoon is employed to gather a standardized portion (approximately 0.25 g) of feces from multiple points. Subsequently, the sampling spoon is securely replaced within the container, and the container is sealed before being it for immediate testing. The Automated Fecal Processing Analysis System (FA180) automatically introduces the collected sample into the sample collection tube, performs mixing procedures, captures an image of the resultant reaction, and facilitates its interpretation. One qualifying FOBT was performed for each patient using one kit, and a patient's FOBT result was considered positive when a hemoglobin level of ≥ 0.2 μg/mL was achieved.

All covariates were extracted from medical records, encompassing demographic details, characteristics of the adenomas, comorbidities, and laboratory parameters. Comprehensive specifics can be found in Table 1. Data on the morphology, size, and location of the adenoma were retrieved from the endoscopic report; pathological features of the adenoma, such as a high degree of dysplasia, chorionic component, and combined non-advanced adenoma, were retrieved from the histopathological report. Marital status, alcohol consumption, and smoking status were categorized by the delineations provided in our prior study^33^. Laboratory indicators were derived from the initial test results documented during the hospitalization period.

### Statistical analysis

Continuous data were presented as either mean ± standard deviation or as median values accompanied by the interquartile range. Statistical assessment of continuous variables involved the utilization of either the Mann–Whitney *U* test or the Student's t-test, contingent upon the data's distribution. Categorical variables were subjected to analysis via either the chi-squared test or the Fisher's exact test. The calculation of 95% confidence intervals for sensitivity was executed using the Clopper–Pearson method. The chi-square test was employed to evaluate the statistical significance of FOBT sensitivity concerning distinct adenoma characteristics.

Logistic regression models were employed to explore the interrelation between the location, size, and pedunculated type of the AA and the occurrence of FOBT false-negative results. The covariates incorporated into the multivariable logistic regression models encompassed those that exhibited significant differences in univariate analyses, those substantiated by precedent scientific evidence, and covariates resulting in effect estimate fluctuations surpassing 10%. We constructed three models: (1) unadjusted; (2) adjusted for sex and age; and (3) adjusted for sex, age, weight, smoking, drinking, antiplatelet anticoagulant usage, size, location, pedunculated type, high-grade dysplasia, villous component, platelets (PLT), hemoglobin (HGB), triglyceride (TG), family history of CRC, hypertension, HLP, and DM. Stratified binary logistic regression models alongside interaction testing, were applied for subgroup analyses. In the study, the covariates with missing data were weight (1.46%), PLT (1.75%), HGB (1.75%), TC (15.50%), TG (15.50%), LDL (15.50%), and HDL (15.50%). Multiple imputations utilizing five replications and a chained equation approach as part of the R mice procedure.

The Clopper-Pearson method was applied using SPSS 25 software. Additional data analyses were conducted using two distinct statistical software platforms: R version 3.3.2 (http://www.R-project.org, The R Foundation) and Free Statistics Software version 1.7.1. A significance threshold of *P* < 0.05, with a two-tailed test, was considered indicative of statistical significance.

### Supplementary Information

Supplementary Legends.

Supplementary Figure S1.

Supplementary Table S1.

## Data Availability

The datasets analyzed during the current study are available from the corresponding author upon reasonable request.

## Abbreviations

FOBT: Fecal occult blood test
AA: Advanced adenoma
CRC: Colorectal cancer
PLT: Platelets
HGB: Hemoglobin
TG: Triglyceride
TC: Total cholesterol
HDL: High-density lipoprotein
LDL: Low-density lipoprotein
HLP: Hyperlipidemia
DM: Diabetes mellitus
HLP: Hyperlipidemia
DM: Diabetes mellitus
CI: Confidence interval
OR: Odds ratio
