# Supplementary material for: Adenoma location, size, and morphology are risk factors for FOBT false-negative results in inpatients with advanced colorectal adenoma
Source: Sci Rep. 2024 Jan 8;14:831. doi: 10.1038/s41598-024-51377-0 (PMC10774257; doi:10.1038/s41598-024-51377-0)
Supplement: Supplementary file 1 — Supplementary Legends. [file 41598_2024_51377_MOESM1_ESM.docx]

**Figure S1.** Subgroup analysis between the advanced adenoma size, location, and pedunculated type with FOBT false-negative results among inpatients. Each stratification factor was adjusted for sex, age, weight, smoking, drinking, antiplatelet anticoagulant use, location, pedunculated type, high-grade dysplasia, villous component, TG, PLT, HGB, family history of CRC, hypertension, HLP, and DM except for the variable itself. PLT, platelets; HGB, hemoglobin, TG, triglyceride; CRC, colorectal cancer; HLP, hyperlipidemia; DM, diabetes mellitus.

**Table S1.** Basic characteristics of participants with and without FOBT.
