# Supplementary figures and images for: Adenoma location, size, and morphology are risk factors for FOBT false-negative results in inpatients with advanced colorectal adenoma
Source: Sci Rep. 2024 Jan 8;14:831. doi: 10.1038/s41598-024-51377-0 (PMC10774257; doi:10.1038/s41598-024-51377-0)

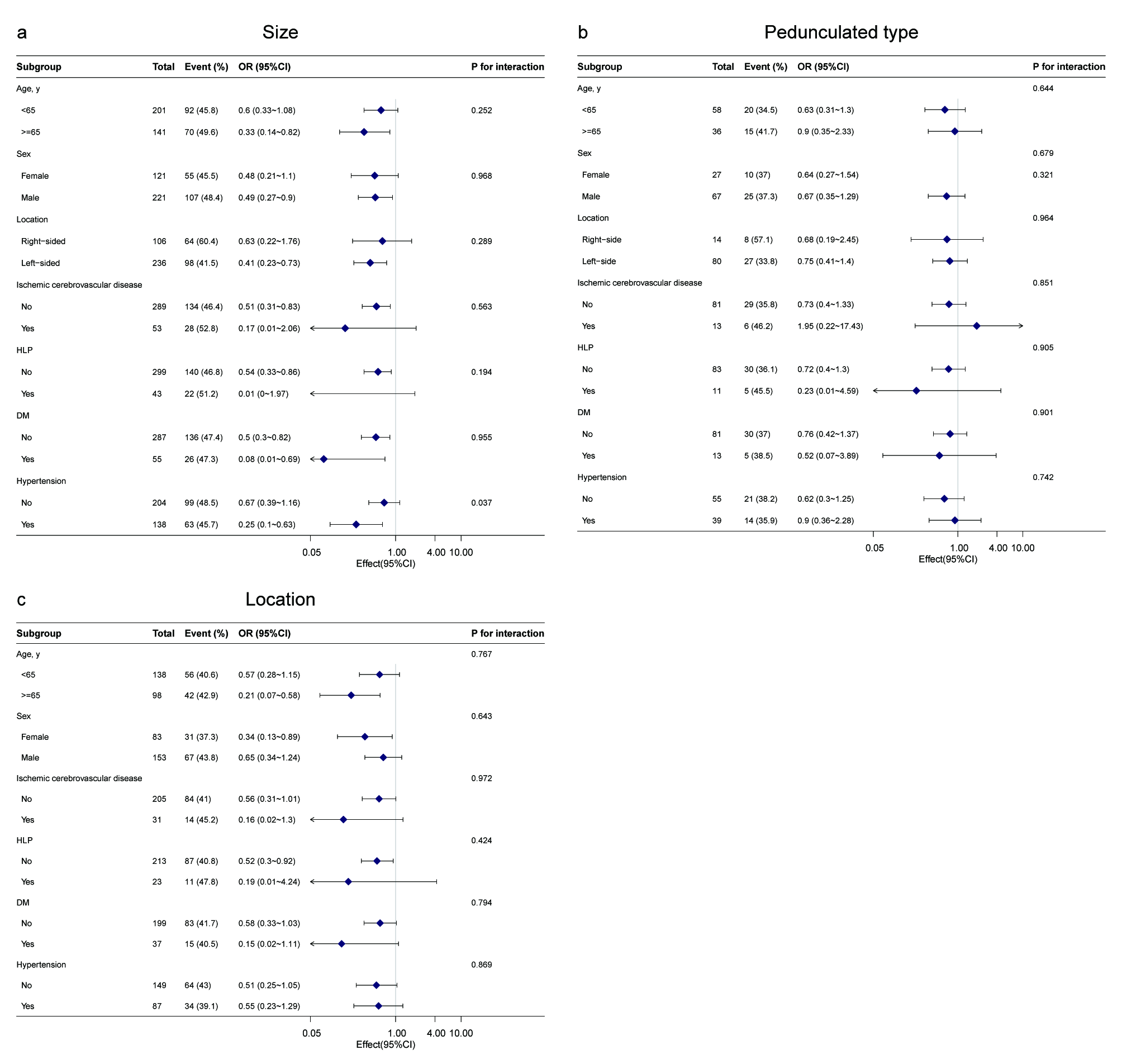

Supplement: Supplementary file 2 — Supplementary Figure S1. [file 41598_2024_51377_MOESM2_ESM.tif]
