# Supplementary material for: Adenoma location, size, and morphology are risk factors for FOBT false-negative results in inpatients with advanced colorectal adenoma
Source: Sci Rep. 2024 Jan 8;14:831. doi: 10.1038/s41598-024-51377-0 (PMC10774257; doi:10.1038/s41598-024-51377-0)
Supplement: Supplementary file 3 — Supplementary Table S1. [file 41598_2024_51377_MOESM3_ESM.docx]

Table S1. Basic characteristics of participants with and without FOBT.

| Variables | Total  n = 572 | With FOBT  n = 342 | Without FOBT  n = 230 | *P*-value |
| --- | --- | --- | --- | --- |
| Age, year | 61.4 ± 10.2 | 61.9 ± 9.7 | 60.5 ± 10.9 | 0.110 |
| Sex, n (%) | 367 (64.2) | 221 (64.6) | 146 (63.5) | 0.780 |
| Marital status, n (%) |  |  |  | 0.269 |
| Single/ divorced | 15 (2.6) | 12 (3.5) | 3 (1.3) |  |
| Married | 527 (92.1) | 312 (91.2) | 215 (93.5) |  |
| Others | 30 (5.2) | 18 (5.3) | 12 (5.2) |  |
| Weight, kg | 70.3 ± 11.8 | 70.0 ± 11.7 | 70.8 ± 12.0 | 0.467 |
| Family history of CRC, n (%) | 10 (1.7) | 4 (1.2) | 6 (2.6) | 0.211 |
| Smoking status, n (%) |  |  |  | 0.073 |
| Non-smoker | 329 (57.5) | 207 (60.5) | 122 (53) |  |
| Current-smoker | 53 (9.3) | 34 (9.9) | 19 (8.3) |  |
| NA | 190 (33.2) | 101 (29.5) | 89 (38.7) |  |
| Drinking status, n (%) |  |  |  | 0.028 |
| Non-drinker | 328 (57.3) | 203 (59.4) | 125 (54.3) |  |
| Current-drinker | 55 (9.6) | 39 (11.4) | 16 (7) |  |
| NA | 189 (33.0) | 100 (29.2) | 89 (38.7) |  |
| Pedunculated type, n (%) | 149 (26.0) | 94 (27.5) | 55 (23.9) | 0.340 |
| With non-advanced adenoma, n (%) | 508 (88.8) | 306 (89.5) | 202 (87.8) | 0.540 |
| Location, n (%) |  |  |  | 0.766 |
| Right-sided | 180 (31.5) | 106 (31) | 74 (32.2) |  |
| Left-sided | 392 (68.5) | 236 (69) | 156 (67.8) |  |
| Largest adenoma size, mm | 1.4 ± 0.6 | 1.4 ± 0.6 | 1.4 ± 0.6 | 0.888 |
| With villous component, n (%) | 129 (22.6) | 79 (23.1) | 50 (21.7) | 0.703 |
| With high grade dysplasia, n (%) | 73 (12.8) | 43 (12.6) | 30 (13) | 0.869 |

Data are presented as the N (%), median (quartile 1–quartile 3), or mean ± SD.

Abbreviation: NA, not recorded.
